# Antidiabetic Potentiality of the Aqueous-Methanolic Extract of Seed of *Swietenia mahagoni* (L.) Jacq. in Streptozotocin-Induced Diabetic Male Albino Rat: A Correlative and Evidence-Based Approach with Antioxidative and Antihyperlipidemic Activities

**DOI:** 10.1155/2011/892807

**Published:** 2010-09-28

**Authors:** Debasis De, Kausik Chatterjee, Kazi Monjur Ali, Tushar Kanti Bera, Debidas Ghosh

**Affiliations:** ^1^Department of Bio-Medical Laboratory Science and Management, (U.G.C Innovative Department), Vidyasagar University, Midnapore 721 102, West Bengal, India; ^2^Pharmaceutical Division, Southern Health Improvement Samity (SHIS), South 24 Paraganas, Bhangar 743 502, West Bengal, India

## Abstract

Antidiabetic, antioxidative, and antihyperlipidemic activities of aqueous-methanolic (2 : 3) extract of *Swietenia mahagoni* (L.) Jacq. (family Meliaceae) seed studied in streptozotocin-induced diabetic rats. Feeding with seed extract (25 mg 0.25 mL distilled water^−1^100 gm b.w.^−1^rat^−1^ day^−1^) for 21 days to diabetic rat lowered the blood glucose level as well as the glycogen level in liver. Moreover, activities of antioxidant enzymes like catalase, peroxidase, and levels of the products of free radicals like conjugated diene and thiobarbituric acid reactive substances in liver, kidney, and skeletal muscles were corrected towards the control after this extract treatment in this model. Furthermore, the seed extract corrected the levels of serum urea, uric acid, creatinine, cholesterol, triglyceride, and lipoproteins towards the control level in this experimental diabetic model. The results indicated the potentiality of the extract of *S. mahagoni* seed for the correction of diabetes and its related complications like oxidative stress and hyperlipidemia. The extract may be a good candidate for developing a safety, tolerable, and promising neutraceutical treatment for the management of diabetes.

## 1. Introduction

Diabetes mellitus is a multifarious group of symptoms characterized by hyperglycemia, abnormal lipid and protein metabolism, along with specific long-term complications affecting the retina, the kidney, and the nervous system mainly [1]. Consumption of calorie-rich diet, obesity, and sedentary life style have lead to tremendous increase in the number of diabetics worldwide especially in Asia [2]. Many oral hypoglycaemic agents, such as sulfonylurea and biguanides, are available along with insulin for the treatment of diabetes mellitus, but these agents have significant side effects [3], and some are ineffective in chronic diabetic patients [4]. Thus, there is an increasing demand of new antidiabetic natural products especially neutraceuticals with lesser side effects and high antidiabetic potential. 

In this context, worldwide efforts have been taken to improve plant-based therapies [5]. WHO [6] recommended for the assessment of traditional medicinal plant in connection with the management of diabetes mellitus [7–9]. Currently, several hundred plants have been reported to have beneficial effects for the treatment of diabetes mellitus, and we have several reports in this line [10–12] as well as of others [13–15]. Research on phytomolecules as diabetic remedies is upraising gradually as these are with minimal or no side effects [16–18]. *Swietenia mahagoni *(*S. mahagoni*), is under family Meliaceae, beautiful, lofty, evergreen, large native tree of tropical America, Mexico, South America, and India. Usually, this plant is 30–40 meters in height and 3-4 meters in girth [19]. The seeds of *S. mahagoni* have been reported for its anti-inflammatory, antimutagenecity, and antitumour activities [20]. In Indonesia and in India, *S. mahagoni *seed used as folk medicine to cure diabetes [21]. There is no systematic work about the antidiabetic activity of *S. mahagoni* though there are very few informations of this plant in this line [22, 23]. The present study was therefore carried out to evaluate the traditional used of *S. mahagoni *as antidiabetic scientifically. Furthermore, the positive roles of natural products (neutraceuticals) for the correction of oxidative stress and hyperlipidaemia, which are diabetes-related complications, were also assessed.

## 2. Materials and Methods

### 2.1. Preparation of Seed Extract

*Swietenia mahagoni* seeds were collected from Midnapore, District Paschim Midnapore, West Bengal, India, in the month of December and were identified by taxonomist of Botany Department, Vidyasagar University, Midnapore. A voucher specimen was submitted in the Department of Botany, Vidyasagar University and numbered as *Swietenia mahagoni *(L.) Jacq./VU/01/09.

Seeds were dried in an incubator for 2 days at 40°C, crushed in an electric grinder, and then pulverized. Out of this powder, 50 g was suspended in the mixture solvent consisting of 80 mL of water and 120 mL methanol and the mixture was kept in an incubator at 37°C for 36 hours. The mixture was stirred intermittently for a 4-hours interval. The mixture was then filtered and filtrate was dried under low pressure and low temperature using rotary evaporator fitted with vacuum pump. Finally, 3.2 gm of powder was collected. This was discovered in distilled water in a fixed dose and used for the treatment.

### 2.2. Chemicals

Streptozotocin (STZ) was obtained from Sigma (USA). All other chemicals used here were of analytical grade obtained from E. Merck, Mumbai, and HIMEDIA, Mumbai, India or purchased from Sigma-Aldrich Diagnostic Ltd. USA. Kits for different enzyme assay were purchased from Crest Biosystems, Goa, India.

### 2.3. Selection of Animal and Animal Care

Twenty four matured normoglycemic (having fasting blood glucose level 80–90 mg/dL) Wistar strain male albino rats, 3 months of age, weighing about 120 ± 10 g were selected for this experiment. Animals were acclimated for a period of 15 days in our laboratory condition prior to the experiment. Rats were housed at an ambient temperature of 25 ± 2°C with 12 hours light : 12 hours dark cycle. Rats were fed pellet diet and water *ad libitum*. The principle of Laboratory Animal Care and instructions given by our Institutional Ethical Committee were followed throughout the experiment.

### 2.4. Induction of Diabetes in Rats

Twenty-four hours fasted eighteen rats out of twenty four were subjected to a single intramuscular injection of STZ (4 mg 100^−1^ b.w) in 0.1 mL of citrate buffer (pH = 4.5) 100 g^−1^, b.w.^−1^rat^−1^. After 7 days of STZ injection, diabetic rats (fasting blood glucose level >250 mg/dL <350 mg/dL) were selected for the study.

### 2.5. Animal Treatment

Twelve diabetic rats having said criteria were selected. Six rats were categorized into diabetic control and the rest of rats were placed in extract administered diabetic group. Other six normoglycemic rats were considered under control group. Extract treatment of *S. mahagoni* seed was started from the 7th day of postinjection period of STZ and was considered as 1st day of experiment. The treatment was continued for next 21 days. 

Group I (control group). Rats of this group received single intramuscular injection of citrate buffer (0.1 mL 100 g^−1^ b.w.^−1^) at the time of STZ injection to the other animals for diabetic induction.

Group II (diabetic control group). Diabetic rats of this group were forcefully fed with water at a dose of 0.25 mL of distilled water 100 g b.w^−1^day^−1^ for 21 days by gavage.

 Group III (extract administered diabetic group). Diabetic rats of this group were forcefully fed with aqueous-methanolic (2 : 3) extract of *S. mahagoni* seed at a dose of 25 mg 0.25 mL water^−1^100 g b.w.^−1^day^−1^ for 21 days at fasting state by gavage.

Extract administration to the rats of group III was performed early in the morning and at fasting state by gavage. Animals of the control group (Group I) were subjected to gavage of distilled water like group II for 21 days at the time of extract treatment to the animals of group III to keep all the animals under the same experimental condition and stress imposition if any due to treatment of extract and animal handling. Starting from first day of extract treatment to diabetic rats, fasting blood glucose levels (12 hours after feed delivery) in all the groups were measured by single touch glucometer on every 7-day interval. On the 21st day of experiment, blood was collected from the tail vein, and fasting glucose level was monitored by single touch glucometer. All the animals were sacrificed at fasting state by light ether anesthesia followed by decapitation after recording the final body weight. Blood was collected from the dorsal aorta by a syringe and the serum was separated by centrifugation at 5000 rpm for 5 minutes for the estimation of serum toxicity study. The liver, kidney, and skeletal muscles were dissected out and stored at −20°C for the quantification of glycogen, for the assessment of the activities of the antoxidant enzymes—catalase (CAT) and peroxidase (Px), and for the quantification of the levels of the products of free radicals like conjugated diene (CD) and thiobarbituric acid (TBARS). Assessment of protein metabolic and lipid metabolic disorders was also performed by the measurement of the levels of serum urea, uric acid, creatinine, total cholesterol, triglyceride, high density, and low-density and very low-density lipoprotein cholesterol.

### 2.6. Estimation of Glycogen Level

Hepatic glycogen level was measured according to the standard protocol [24]. In brief, hepatic tissues was homogenized in hot ethanol (80%) at a tissue concentration of 100 mg mL^−1^ and then centrifuged at 9500 rpm for 20 minutes. The residue was collected, dried over a water bath, and then extracted at 0°C for 20 minutes by adding a mixture of 5 mL water and 6 mL of 52% perchloric acid. The collected material was centrifuged at 9500 rpm for 15 minutes for recovery of the supernatant. From the recovered part, 0.2 mL supernatant was transferred in graduated test tube and made to 1 mL volume by the addition of distilled water. Graded standards were prepared using 0.1, 0.2, 0.4, 0.6, 0.8, and 1.0 mL of a working standard solution, and volume of all each standard solution was made to 1 mL using distilled water. Anthrone reagent (4 mL) was added to all the test tubes and the tubes, were then heated in a boiling water bath for 8 minutes, allowed to cool at room temperature, and the intensity of the green to dark green color of the solution was recorded at 630 nm. Glycogen content of the sample was determined from a standard curve prepared with standard glucose solution.

### 2.7. Biochemical Assay of Antioxidant Enzymes

The activities of catalase of the liver, kidney, and skeletal muscles were measured biochemically [25]. For the evaluation of catalase activity, target organ of each animal was homogenized separately in 0.05 M Tris-HCl buffer solution (pH-7.0) at the tissue concentration of 50 mg mL^−1^. These homogenized samples were centrifuged at 10,000 rpm at 4°C for 10 minutes. In spectrophotometric cuvette, 0.5 mL of 0.00035 M H_2_O_2_ and 2.5 mL of distilled water were mixed and reading of absorbance was noted at 240 nm. Supernatant of sample was added at a volume of 40 *μ*L and the subsequent six readings were noted at 30-second interval.

The peroxidase activity was measured in the above-said tissues, according to the standard method [26]. The samples were homogenized in ice-cold of 0.1 M phosphate buffer saline (pH-7.0) at the tissue concentration of 50 mg mL^−1^. Next, 20 mM guiacol was mixed with 0.1 mL supernatant collected from the homogenate. In presence of 0.3 mL of 12.3 mM H_2_O_2_, the time was recorded for an increase in the absorbance by 0.1 at 436 nm.

### 2.8. Measurement of Protein Metabolic Disorders

#### 2.8.1. Serum Urea, Uric acid, and Creatinine

Levels of serum urea, uric acid, and creatinine were measured using kits from Merck Diagnostic Ltd, India [27–29], following spectrometric methods. The values were expressed in mg dL^−1^ in all the cases.

### 2.9. Measurement of Lipid Metabolic Disorders

#### 2.9.1. Serum Total Cholesterol (TC), Lipoprotein Cholesterol, and Triglyceride (TG)

Serum TC was quantified spectrophotometrically [30] by the addition of enzyme present in the reagent kit (Span Diagnostic Ltd, Surat, India). The absorbance of red quinoneimine complex was determined at 505 nm. The value of TC present in serum was expressed in mg dL^−1^.

Levels of serum low-density lipoprotein cholesterol (LDLc) and very low-density lipoprotein cholesterol (VLDLc) were measured according to a standard protocol [31]. High-density lipoprotein cholesterol (HDLc) level was measured biochemically [32].

Serum TG level was measured by using a kit from Span Diagnostics Pvt. Ltd, Boroda, India. The absorbance was measured at 520 nm. The value was expressed in mg dL^−1^ [33].

### 2.10. Quantification of Lipid Peroxidation from Concentration of Thiobarbituric Acid Reactive Substance (TBARS) and Conjugated Diene (CD) in Liver, Kidney, and Skeletal Muscles

The above mentioned tissues were homogenized separately at the concentration of 50 mg mL^−1^ in 0.1 M of ice-cold phosphate buffer (pH-7.4) and the homogenates were centrifuged at 10,000 rpm at 4°C for 5 min individually. Each supernatant was used for the estimation of TBARS and CD levels. For the quantification of TBARS, the homogenized mixture of 0.5 mL was mixed with 0.5 mL of normal saline (0.9 g % NaCl) and 2 mL of TBA-TCA mixture (0.392 g thiobarbituric acid in 75 mL of 0.25 N HCl with 15 g trichloroacetic acid). The volume of the mixture was made up to 100 mL by 95% ethanol and boiled at 100°C for 10 minutes. This mixture was then cooled at room temperature and centrifuged at 4000 rpm for 10 minutes. The whole supernatant was taken in spectrophotometer cuvette, and absorbance was read at 535 nm [34]. Quantification of the CD was performed by a standard method [35]. In brief, the lipids from the homogenate were extracted with chloroform-methanol (2 : 1) mixture followed by centrifugation at 1000 rpm for 5 min. The chloroform layer was evaporated to dryness under a stream of nitrogen. The lipid residue was dissolved in 1.5 mL of cyclohexane and the absorbance was noted at 233 nm to measure the amount of hydroperoxide formed.

### 2.11. Statistical Analysis

Analysis of variance (ANOVA) followed by multiple comparison two-tail “*t*” test was used for statistical analysis of collected data [36]. Differences were considered significant at *P* < .05. All the values were indicated in the figures as Mean ± S.E.M (Standard Error of Mean).

## 3. Results

### 3.1. Blood Glucose Level

Diabetes induced by STZ resulted in a significant elevation in blood glucose level in comparison to the control group. After administration of aqueous-methanolic (2 : 3) extract of *S. mahagoni *seed to the diabetic animals for 21 days, a significant reduction in blood glucose level was noted which was close to the control level (Table 1).

### 3.2. Hepatic Glycogen Level

**Figure 1:**
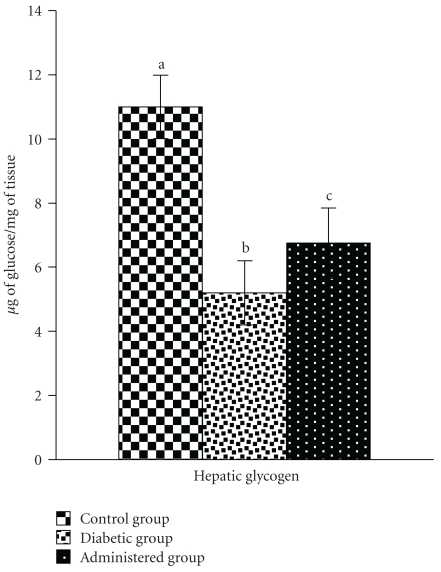
Correction of glycogen content in hepatic tissue after administration of aqueous-methanolic extract of *S. mahagoni *seed in STZ-induced diabetic male albino rat. Each bar represents Mean ± S.E.M; *n* = 6. ANOVA followed by multiple comparison two tail “*t*” test. Bars diagrams with different superscripts (a,b,c) differ from each other significantly, *P* < .05.

Hepatic glycogen content was decreased in the diabetic control group in comparison with the control group. After treatment of this herbal extract to the diabetic animals, there was a significant recovery in the glycogen content towards the control level (Figure 1).

### 3.3. Activities of CAT and Px

**Figure 2:**
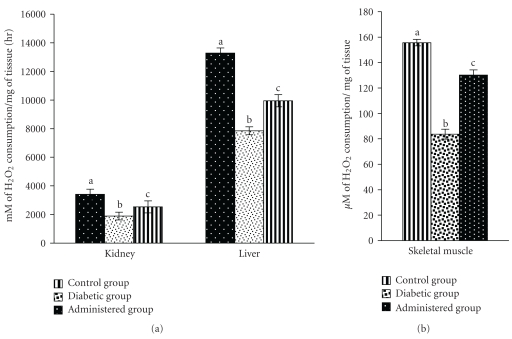
Resettlement in the activities of peroxidase in kidney, liver, and skeletal muscles after administration of aqueous-methanolic extract of *S. mahagoni* seed in STZ-induced diabetic male albino rat. Bar expressed as Mean ± S.E.M; *n* = 6. ANOVA followed by multiple comparison two-tail “*t*”-test. Bars with different superscripts (a,b,c) differ from each other significantly, *P* < .05.

**Figure 3:**
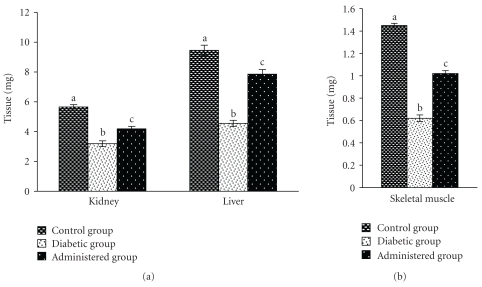
Effect of aqueous-methanolic extract of *S. mahagoni* seed on the activities of catalase in kidney, liver, and skeletal muscles in STZ-induced diabetic male albino rat. Bar represents Mean ± S.E.M; *n* = 6. ANOVA followed by multiple comparison two-tail “*t*”-test. Bars with different superscripts (a,b,c) differ from each other significantly, *P* < .05.

Activities of CAT and Px in liver, kidney, and skeletal muscles were decreased significantly in diabetic control group with respect to the control group. After the treatment of aqueous-methanolic (2 : 3) extract of *S. mahagoni* seed to STZ-induced diabetic rat, the levels of above enzyme activities were resettled towards the control level (Figures 2 and 3).

### 3.4. Serum Urea, Uric acid, and Creatinine Levels

**Figure 4:**
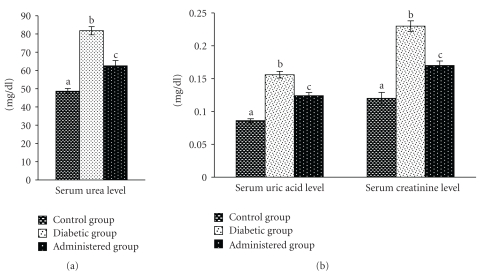
Correction in the levels of urea, uric acid, and creatinine in serum after administration of aqueous-methanolic extract of *S. mahagoni* seed in STZ-induced diabetic male albino rat. Bar represents Mean ± S.E.M; *n* = 6. ANOVA followed by multiple comparison two-tail `*`* *t*”-test. Bars with different superscripts (a,b,c) differ from each other significantly, *P* < .05.

Serum urea, uric acid, and creatinine levels were increased significantly in the diabetic control group with respect to the control group. The levels of these parameters were restored towards the control level after administration of aqueous-methanolic extract of the seeds of *S. mahagoni* to the diabetic rat (Figure 4).

### 3.5. Serum Lipid Profile

**Figure 5:**
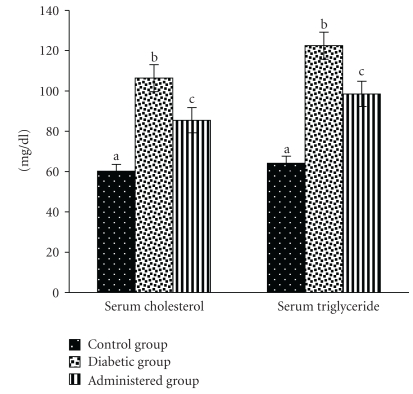
Correction in the levels of total cholesterol and triglyceride in serum after treatment of aqueous-methanolic extract of *S. mahagoni* seed in STZ-induced diabetic male albino rat. Bar are expressed as Mean ± S.E.M; *n* = 6. ANOVA followed by multiple comparison two-tail “*t*”-test. Bars with different superscripts (a,b,c) differ from each other significantly, *P* < .05.

Serum total cholesterol (TC) and triglyceride (TG) levels were significantly elevated in the diabetic control group in comparison with the control group. After treatment with the above-mentioned extract to the diabetic animals, serum TC and TG levels were recovered significantly towards the control level (Figure 5).

**Figure 6:**
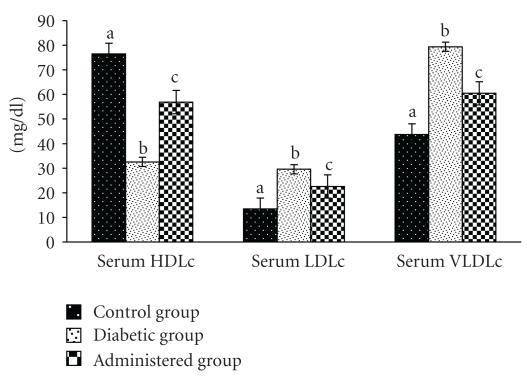
Effect of aqueous-methanolic extract of *S. mahagoni* seed on serum high density lipoprotein cholesterol (HDLc), low density lipoprotein cholesterol (LDLc) and very low density lipoprotein cholesterol (VLDLc) levels in STZ-induced diabetic rat. Bar represents Mean ± S.E.M; *n* = 6. ANOVA followed by multiple comparison two-tail “*t*”-test. Bars with different superscripts (a,b,c) differ from each other significantly, *P* < .05.

Other parameters of this lipid profile like serum LDLc and VLDLc levels were elevated and serum HDLc level was decreased in the diabetic control group in respect to the control. The levels of the above-mentioned parameters were recovered significantly towards the control group after treatment of the extract of *S. mahagoni* seed when compare with the diabetic control group (Figure 6).

### 3.6. Levels of CD and TBARS

**Figure 7:**
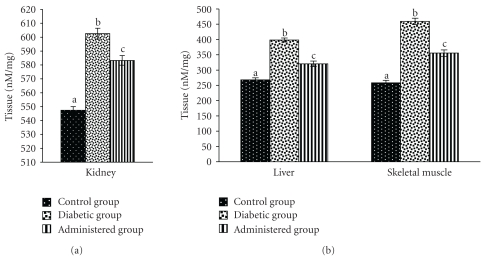
Effect of aqueous-methanolic extract of *S. mahagoni* seed on the levels of conjugated diene (CD) in kidney, liver, and skeletal muscles in STZ-induced diabetic male albino rat. Bar represents Mean ± S.E.M; *n* = 6. ANOVA followed by multiple comparison two-tail “*t*”-test. Bars with different superscripts (a,b,c) differ from each other significantly, *P* < .05.

**Figure 8:**
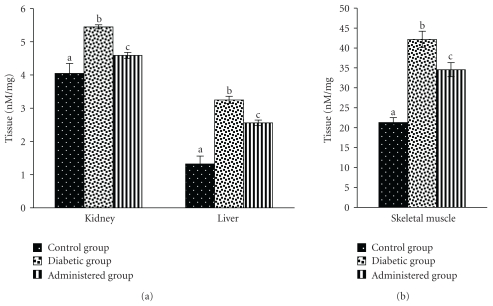
Correction in the levels of thio-barbituric acid reactive substances (TBARS) in kidney, liver, and skeletal muscles after administration of aqueous-methanolic seed extract of *S. mahagoni* in STZ-induced diabetic male albino rat. Values expressed as Mean ± S.E.M; *n* = 6. ANOVA followed by multiple comparison two-tail “*t*”-test. Bars with different superscripts (a,b,c) differ from each other significantly, *P* < .05.

Levels of CD and TBARS in liver, kidney, and skeletal muscles were increased significantly in the diabetic control group when compared to the control group. Significant recovery was noted in the levels of the above-mentioned parameters in liver, kidney and skeletal muscles after administration of the seed extract to the diabetic animal (Figures 7 and 8).

## 4. Discussion

**Figure 9:**
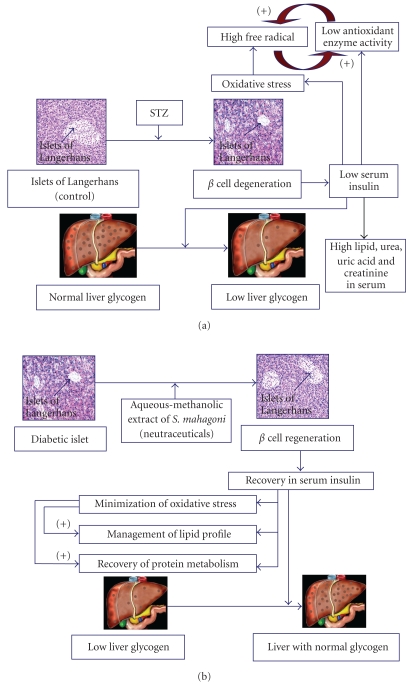
Diagrammatic representation of diabetes induction by STZ (a) and the hypothetical ways the correction of diabetes-induced metabolic disorders by aqueous-methanolic extract of *S. mahagoni* (b).

The present study focuses the antidiabetic, antihyperlipidemic, and antioxidative capacities, as well as protein metabolic disorders management efficacy of the aqueous-methanolic extract (2 : 3) of *S. mahagoni* seed in STZ-induced diabetic male albino rat. The pilot studies focused on the fact that the aqueous-methanol (2 : 3) extract was the most effective studied here out of the other extracts, for the correction of above said disorders in STZ-induced diabetic rat. Here, metabolic disorders in STZ-induced diabetic rat have been established by the levels of blood glucose, hepatic glycogen, serum urea, uric acid, creatinine, cholesterol, triglyceride, and lipoproteins. These results are in the same line of our previous studies [10–12, 37] and of others [38, 39]. Oxidative stress developed in diabetic state is in parallel to our previous reports and also in agreement with others [11, 12, 40–43]. Oxidative stress in diabetic model has been focused here by the assessment of CAT and Px activities in liver, kidney, and skeletal muscles, important biosensors for oxidative stress assessment [44, 45]. Diabetes-induced oxidative stress has been confirmed here by the elevation in the levels of end products of free radicals, that is, TBARS and CD, indicators of oxidative injury [46, 47]. Diabetes-associated oxidative stress is developed by many biochemical pathways such as glucose autoxidation, protein glycation, and so forth [48]. In diabetes, protein catabolism is increased due to deficiency of carbohydrate-derived energy in connection with low-serum insulin [49]. This has been indicated here by high levels of serum urea, uric acid, and creatinine. High-serum creatinine level is also the marker of muscle wastage [50]. All these metabolic disorders in STZ-induced diabetic rat were represented here by line diagram (Figure 9(a)). Glycemic controlling capacity of the extract in STZ induced diabetic state has been supported here by the correction of blood glucose, and glycogen content in liver, important sensors in this concern [51]. The above-mantioned correction may be due to insulin mimetic action of the above-mantioned extract as insulin is one of the important regulators of glycogen synthesis [52]. Correction of oxidative injury which is associated with diabetes [53] is another possibility of the recovery in glycemic disorders. The plant extract was able to recover the protein metabolic disorders possibly by stimulating the existing *β* cells and or by regenerating *β* cells like other plant products which have been claimed by us [40] as well as by others [15, 51]. 

Hyperlipidemia is associated with diabetic state [54] and this may be due to uninhibited action of lipase [55]. High levels of serum cholesterol, triglyceride, LDLc, and VLDLc along with low level of serum HDLc in STZ-induced diabetic state focused the low level of serum insulin and the results are consistence to our previous findings [11] and of others [56]. Since insulin inhibits adipose tissue hormone sensitive lipase and reduces lipolysis, the aqueous-methanolic extract of *S. mahagoni* seed may correct the above mantioned disorders by mimicking insulin action. The most exciting results and the additional advantage of this extract over the existing drugs in this concern is the correction of triglyceride and elevation in HDLc level as the most of the drugs those decreased the blood level of triglyceride also decreased the level of HDLc [57]. High level of triglyceride and low level of HDLc are independently related to morbidity and deaths in diabetic subjects by the induction of to coronary heart diseases [58, 59]. 

The extract is able to correct the diabetes-induced oxidative injury which has been supported here by the elevation in the activities of antioxidant enzymes and diminution in the quantity of the products of the free radicals. This correction may be due to the antidiabetic efficacy of this extract that prevents the reactive oxygen species generation by preventing glucose autooxidation and by glycation. Another possibility is the presence of antioxidative types of neutraceutical like flavonoids in the above-mantioned extract. 

From the above results, the antidiabetic potentiality of aqueous-methanolic extract of *S. mahagoni* seed may be explained by two ways. One way may be the insulinotrophic effect of this extract that results correction in blood glucose level, glycogen level in liver, the levels of serum lipid profile, and bio-sensors of protein metabolism as all of these are under the control of serum insulin [60–62]. Another way may be the oxidative stress protection which is developed mainly in metabolic tissues in diabetes. This has been reflected here by the correction of antioxidant enzyme activities that lowered the levels of end products of free radicals. These antioxidant activities also protect the metabolic enzymes in cells that resettled the cellular homeostasis towards the normal level. The hypothetical view for the corrective effect of the plant extract on STZ-induced diabetic hyperglycemia, hyperlipidemia, oxidative injury, and high-protein catabolism may be expressed by the diagram (Figures 9(a) and 9(b)). The specific bioingredient(s) or neutraceuticals present in the extract responsible for such antidiabetic activity cannot be detected but this is under our observation and would be focused from future work in this line.

## 5. Conclusion

In conclusion, it may be stated that the aqueous-methanolic extract of *S. mahagoni* seed may provide a new therapeutic avenue against diabetes and diabetes-related complications. Moreover, further work is necessary to search out the active ingredients present in this extract having antidiabetic efficacy. Extensive research is currently taking place in India, China, and Korea and in other countries in order to develop potential herbal medicine to prevent metabolic diseases including diabetes and its related complications.

## Figures and Tables

**Table 1:** Effect of aqueous-methanol extract of *S. mahagoni *seed on fasting blood glucose level in streptozotocin-induced diabetic male albino rat.

Group	Fasting blood glucose level (mg/dL)					
	0 day	1st day	8th day	15th day	22nd day	29th day
	(Day of STZ administration)		(2nd day of extract treatment)			(21st day of extract treatment)
Control group	73.58 ± 5.2^a^	74.72 ± 4.8^a^	73.86 ± 4.9^a^	74.34 ± 4.7^a^	73.79 ± 4.6^a^	73.88 ± 4.7^a^
Diabetic group	75.64 ± 4.9^a^	295.21 ± 5.2^b^	318.32 ± 5.8^b^	325.54 ± 6.2^b^	317.00 ± 5.9^b^	322.52 ± 6.5^b^
Administered group	73.61 ± 5.1^a^	297.52 ± 4.5^b^	221.50 ± 4.8^c^	172.67 ± 5.2^c^	109.61 ± 5.3^c^	76.26 ± 4.9^a^

Data are expressed as Mean ± S.E.M; *n* = 6. ANOVA followed by multiple comparison two tail “*t*” test. Values with superscripts (a,b,c) in each vertical column differ from each other significantly, *P* < .05.
